# The alpha and the omega (6 lipids): discovering dietary drivers of heterotopic ossification

**DOI:** 10.1172/JCI210090

**Published:** 2026-09-01

**Authors:** Peyton L. Carpen, Matthew B. Greenblatt

**Affiliations:** 1 Department of Pathology and Laboratory Medicine, Weill Cornell Medical College, New York, New York, USA.; 2 Skeletal Health and Orthopedic Research Program, Hospital for Special Surgery, New York, New York, USA.

## Abstract

Bone formation in soft tissues, known as heterotopic ossification (HO), can occur as a complication of trauma or burn injury and can cause pain and functional limitations in the affected site. HO remains an unmet clinical challenge due to a general lack of specific medical therapies. In this issue of the *JCI*, a study by Moye et al. identified obesity as a risk factor for HO and further found that the association with obesity was driven not by caloric surplus, but instead by dietary omega-6 lipids, which are characteristically elevated in the Western diet. These omega-6 lipids accumulated directly at the incipient HO site, where they served as substrates for prostaglandin E_2_ (PGE_2_), fueling aberrant osteoblast differentiation. Overall, this work provides compelling support for dietary intervention or pharmacologic therapy directed at downstream PGE_2_ signaling as approaches to reduce HO in at-risk patients.

## Introduction

Heterotopic ossification (HO) is the aberrant formation of bone in soft tissues. It is a frequent unwanted accompaniment to a variety of traumatic injuries to the limbs, orthopedic procedures including arthroplasty, traumatic brain and spinal cord injuries, and severe burns (1). These sporadic injury-associated forms of HO are mirrored by a rarer and more severe set of genetic disorders, most prominently including fibrodysplasia ossificans progressiva, a progressive form of HO that occurs due to activating *ACVR1* mutations (2–4).

Notably, the mineralized tissue forming in HO is by definition true bone, as it results from an endochondral ossification sequence, including osteoblasts, osteocytes, and, at least transiently, chondrocytes. By invoking the cellular mechanisms of bone formation, HO is distinguished from the soft tissue dystrophic calcifications that can sometimes be observed in the setting of chronic inflammation, which involve direct deposit of calcium mineral without bone tissue formation (5). Surgical management to excise the heterotopic bone can be applied to symptomatic HO, but it is limited both by the inherent morbidity of the procedure itself and by the possibility of recurrence (6).

This combination of HO’s relatively common occurrence as a complication of a range of injuries or surgical procedures alongside the general lack of specific medical therapies has motivated a search for mechanisms that explain why some individuals are apparently at elevated risk of HO, which might in turn point toward potential therapies. To date, these efforts have largely focused on establishing the critical functions of the innate and adaptive immune systems and downstream cytokine signaling, clearly placing inflammation as an early inciting event in HO pathogenesis (7). To better understand which patients are at risk of HO, a recent effort creatively proposed to study gene expression in rare circulating connective tissue progenitor cells participating in the HO (8). Progenitors entering circulation from the incipient HO site do not re-engraft in the tissues to participate in the ongoing HO; nevertheless, these cells represent a promising means to interrogate induction of gene expression programs that contribute to HO progression and thereby identify patients at high risk.

## Dietary omega-6 lipids as drivers of HO

**Figure 1:**
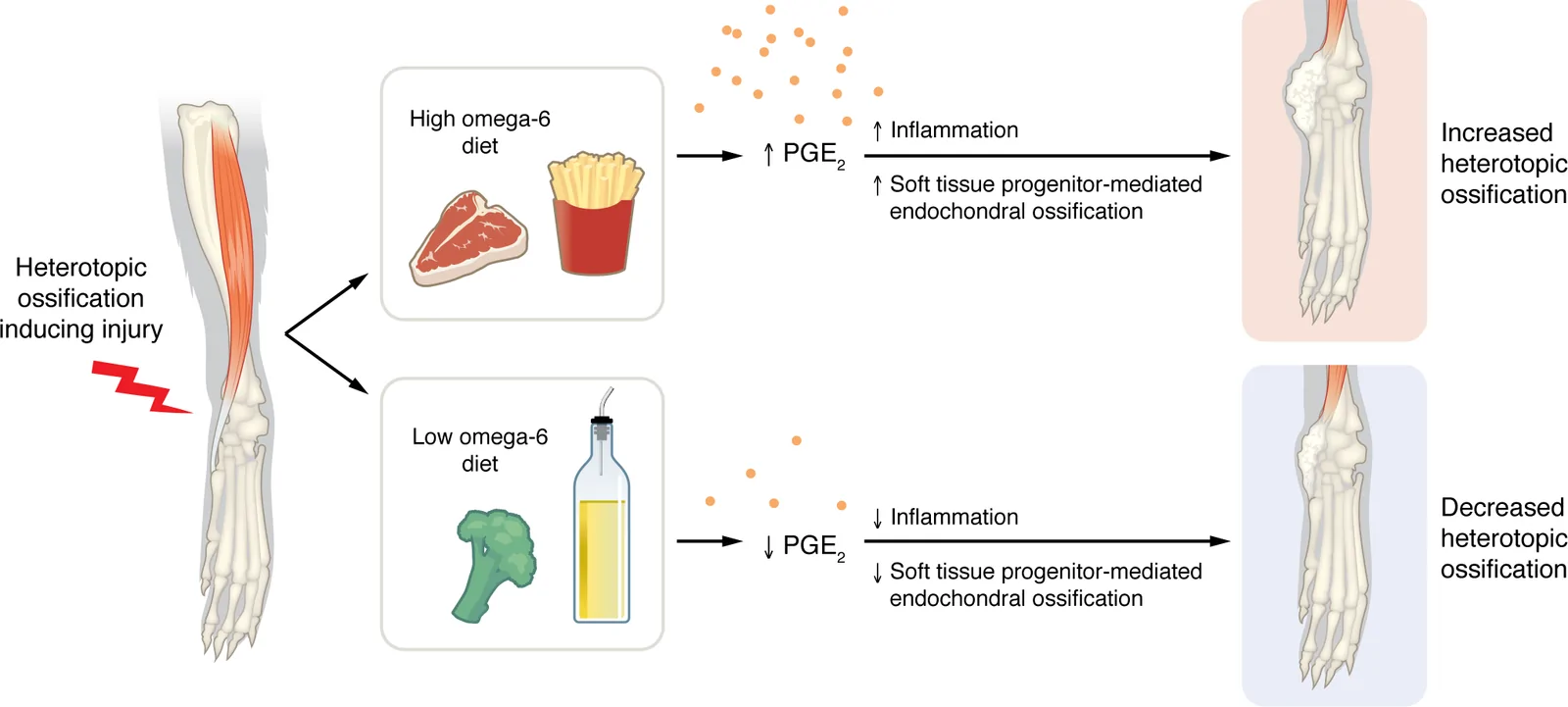
Dietary omega-6 fatty acids drive HO. Moye et al. (9) identified obesity as a risk factor for developing HO, a condition in which bone forms in soft tissue sporadically after limb injury. In a mouse model, depleting dietary intake of omega-6 fatty acids ameliorated HO by reducing local production of PGE_2_, indicating that dietary or pharmacological interventions could be developed for patients at risk of HO.

Extending these efforts, in this issue of the *JCI*, Moye et al. identified dietary omega-6 lipids as drivers of sporadic HO risk (9). Their work builds upon prior retrospective clinical studies finding a link between obesity and HO risk (10). The authors first substantiated this link between obesity and HO, mostly notably by directly establishing that high-fat diet–exposed mice display increased HO in a widely utilized model of burn- and tenotomy-associated HO that arises in tendon tissue. Moye et al. hypothesized that alterations in metabolism likely underlie the dietary effect on HO and performed metabolomics of the incipient HO site. Notably, they observed enrichment of omega-6 polyunsaturated fatty acids, including arachidonic acid and linoleic acid, a precursor for arachidonic acid, in the high-fat diet–exposed animals. This enrichment was coupled with local increases in a range of chemokines and cytokines, including IL-6 and TNF. These omega-6 fatty acids played a causal role in driving HO, as an isocaloric diet with a low omega-6/omega-3 fatty acid ratio had a protective effect in the murine HO model. Importantly, the use of an isocaloric control diet also clarified that this dietary effect on HO is driven directly by omega-6 fatty acids and not by general caloric surplus (Figure 1).

In obesity and in the setting of a high-fat and sugar-enriched Western diet, tissues accumulate omega-6 lipids, which can be converted to proinflammatory eicosanoids, including prostaglandins, thereby altering both immune and connective tissue cellular homeostasis. In particular, the omega-6 lipid arachidonic acid is converted to prostaglandins by the cyclooxygenases COX-1 and COX-2. Moye et al. found that the arachidonic acid enriched at the injury site was converted locally into prostanoids including PGE_2_ via COX-2–expressing myeloid cells. Consistent with past studies indicating that PGE_2_ has osteoanabolic activity, this PGE_2_ acted on local connective tissue progenitors to induce endochondral ossification (11).

In line with COX-2 being a mechanistic link between dietary omega-6 fatty acids and HO, treating the mouse HO model with the COX-2–specific inhibitor celecoxib attenuated HO development. While NSAIDs have been widely studied as a means to prevent HO formation (see ref. 12 for a meta-analysis of these studies), these findings refine the mechanistic context for their activity, placing NSAIDs downstream of a specific dietary pathway driving HO. Moreover, these findings suggest that NSAIDs will be most effective in obese patients at risk of HO, where these dietary omega-6 fatty acid pathways are most active, potentially explaining the observation that NSAIDs are only partially effective in preventing HO development. Moye et al. offer evidence to support this model. Analysis of an aggregated medical records database found that a prescription for celecoxib, a COX-2 inhibitor, significantly reduced HO incidence in obese patients who underwent total joint arthroplasty but did not significantly alter HO incidence in nonobese patients who underwent total joint arthroplasty.

## Dietary interventions to reduce HO risk?

Moye et al.’s study is not only notable for helping to establish the causal role of the proposed omega-6 fatty acid pathway in HO development, it also raises the possibility that dietary interventions may reduce progression to HO in at-risk patients. The concept of dietary interventions for HO is exciting but raises several questions. While there have been prior studies of risk factors contributing to HO development, further work is likely needed to define exactly which at-risk patients would benefit from intensive preventative therapy. Another challenge is that the Moye et al. study frames obesity as largely serving as a proxy for dietary omega-6 fatty acid exposure in the context of evaluating HO risk. This raises the question of whether careful dietary evaluation would be an effective approach for identifying patients at risk of HO, and further pursuit of this hypothesis in clinical studies could represent an important corroboration of the model proposed here. Of course, any dietary intervention must also be evaluated against the convenience and possible patient compliance benefits of simply utilizing the COX-2 inhibitors, which are here proposed to ultimately act in a similar manner.

## Conclusions

Moye et al. established a pathway whereby dietary omega-6 fatty acids are converted to PGE_2_ at sites of incipient HO, thereby inducing local soft tissue progenitors to undertake aberrant endochondral ossification. These findings refine previously proposed links between obesity and HO, proposing that they represent, in part, a specific dietary pathway linking local inflammation to osteogenic differentiation. More broadly, these findings offer insights to the larger question in skeletal biology of why inflammation sometimes appears to promote bone formation, as seen in HO, and sometimes appears to inhibit bone formation, as seen in wide range of disorders including rheumatoid arthritis.

## Conflict of interest

The authors have declared that no conflict of interest exists.

## Funding support

This work is the result of NIH funding, in whole or in part, and is subject to the NIH Public Access Policy. Through acceptance of this federal funding, the NIH has been given a right to make the work publicly available in PubMed Central.

- National Institute of Arthritis and Musculoskeletal and Skin Diseases (R01AR083462 to MBG).
- National Cancer Institute (R01CA282815 and P01CA28525 to MBG).
- Eunice Kennedy Shriver National Institute of Child Health and Human Development (R01HD115274 to MBG).
- Marfan Foundation Innovators award (to MBG).
- Pershing Square Foundation MIND Prize (to MBG).
- NIH/National Center for Advancing Translational Sciences grant TL1TR002386 (to PLC).
- NIH/National Institute of General Medical Sciences, Weill Cornell Medicine Initiative for Maximizing Student Development T32 award (to PLC).

## Version 1 09/01/2026

Electronic publication

## References

[1] Meyers C (2019). Heterotopic ossification: a comprehensive review. JBMR Plus.

[2] Shore EM (2006). A recurrent mutation in the BMP type I receptor ACVR1 causes inherited and sporadic fibrodysplasia ossificans progressiva. Nat Genet.

[3] Yu PB (2008). BMP type I receptor inhibition reduces heterotopic [corrected] ossification. Nat Med.

[4] Chakkalakal SA (2012). An Acvr1 R206H knock-in mouse has fibrodysplasia ossificans progressiva. J Bone Miner Res.

[5] Fournier DE (2020). Dystrophic calcification and heterotopic ossification in fibrocartilaginous tissues of the spine in diffuse idiopathic skeletal hyperostosis (DISH). Bone Res.

[6] Agarwal S (2017). Surgical excision of heterotopic ossification leads to re-emergence of mesenchymal stem cell populations responsible for recurrence. Stem Cells Transl Med.

[7] Ranganathan K (2016). The role of the adaptive immune system in burn-induced heterotopic ossification and mesenchymal cell osteogenic differentiation. J Surg Res.

[8] Nunez J (2026). Early detection of aberrant cell fate and repair using circulating progenitor cells in patients with heterotopic ossification. Nat Commun.

[9] Moye SL (2026). Dietary omega-6 lipids promote postinjury aberrant bone formation in obesity. J Clin Invest.

[10] Qian Y (2019). Obesity may be a risk factor for recurrent heterotopic ossification in post-traumatic stiff elbow among children and teenagers. Orthop Traumatol Surg Res.

[11] Chen H (2019). Prostaglandin E2 mediates sensory nerve regulation of bone homeostasis. Nat Commun.

[12] Yang F (2025). Effectiveness and priority of irradiation and six NSAIDs in prevention heterotopic ossification after total hip arthroplasty: a network meta-analysis of randomized controlled studies. Front Pharmacol.

